# Effect of microhabitat variability on restoration success of swamp willow *Salix myrtilloides* L. population

**DOI:** 10.1038/s41598-025-33807-9

**Published:** 2025-12-30

**Authors:** Aleksander Kołos, Andrzej K. Kamocki, Piotr Banaszuk, Adam Więcko

**Affiliations:** 1 Institute of Forest Sciences, Faculty of Civil Engineering and Environmental Sciences, Białystok University of Technology, Wiejska 45E, 15-351 Białystok, Poland; 2 Institute of Environmental Engineering, Faculty of Civil Engineering and Environmental Sciences, Białystok University of Technology, Wiejska 45E, 15-351 Białystok, Poland; 3 Department of Hydrobiology, Faculty of Biology, University of Białystok, Ciołkowskiego 1J, 15-245 Białystok, Poland

**Keywords:** Restoration effectiveness, Conservation management, Glacial relicts, Wetland biodiversity, Northeastern Poland, Conservation biology, Restoration ecology

## Abstract

Maintaining local populations of rare boreal plant species living at the edge of their geographic ranges is particularly important as the climate continues to warm. In many European countries, their numbers have declined dramatically over the past few decades. In Poland, relict willow species are particularly at risk of extinction, including the swamp willow *Salix myrtilloides*. During a field experiment to reintroduce *S. myrtilloides*, 240 seedlings were planted within the quaking bog surrounding Lake Wiejki on the outskirts of the Knyszyńska Forest (northeastern Poland). Five years after the seedlings were introduced into the wild, the size of the population had increased to 272 individuals, probably due to increased vegetative proliferation caused by the browsing by moose. The flowering efficiency of individuals was initially relatively high (40.82%) but decreased almost twofold (24.27%) between 2019 and 2024. Monitoring over five years indicated that the height of the herb layer and the cover of competing species could affect the height of the aboveground shoots of *S. myrtilloides*. We found a significant statistical relationship between soil water pH and population restoration success, with lower pH promoted vegetative regrowth while limiting flowering.

**Supplementary Information:**

The online version contains supplementary material available at 10.1038/s41598-025-33807-9.

## Introduction

Numerous studies have shown that climate change often leads to significant transformations of peatlands, particularly evident in disturbances to carbon and water storage and the provision of ecosystem services^1–3^. Particularly intense changes are observed in peatlands located in the boreal zone^4^. The most destructive for wetlands are changes in hydrology, which contribute to the depletion or disappearance of numerous associated plant species. Previous studies have shown that boreal species at the edge of their geographic range are particularly vulnerable to extinction^5^.

Poland is one of the most wetland-rich countries in Central Europe. Unfortunately, as in many other European countries, they are subject to intense anthropogenic pressure^6^. Human impact has had a tremendous effect on changes in the habitat and hydrology of peatlands, which in turn entail changes in the floristic structure of plant communities. Endemic and relict plant species are particularly susceptible to such effects. Swamp willow *Salix myrtilloides* L. is one of the rarest glacial relics in Central Europe. Swamp willow is a small shrub of subarctic and boreal origin, reaching a height of 0.2–0.8 m under natural conditions^7^. It inhabits northern, central and eastern Europe, Siberia and much of North America. The southwestern range limit of this species runs through Poland^8^ where *S. myrtilloides* has the status of a rare and endangered species^9^, as in several neighboring countries: Germany, Switzerland, Czech Republic, Slovakia, Lithuania, Ukraine and Belarus^10–16^. The species was once recorded in Poland at about 90 sites scattered in the Mazurian Lake District, Pomeranian Lake District, Western Polesie, Lublin Upland and Lesser Poland Upland, Sudety Mts.^17–19^ of which only a dozen has been confirmed in recent years^9,20–22^. *S. myrtilloides* is disappearing remarkably quickly in the South Pomeranian Lake District^23^, the Masurian Lake District^19^ and Polesie Lublin^24–26^. The leading causes are drainage and peatland use (mowing, grazing, peat extraction), which are followed by the disappearance and transformation of this species’ habitat and increased competition due to the expansion of herbaceous vegetation, trees, and shrubs.

*S. myrtilloides* is found primarily in acidic peatlands on oligo- or mesotrophic soils with pH 3.5–5.5^9^. It is an eminently light-dwelling species and most often grows in sedge communities of the class *Scheuchzerio-Caricetea nigrae*^8,18,24^. This shrub species finds optimal conditions in the *Caricetum limosae* and *Caricetum lasiocarpae* associations^27^. The biocenotic significance of *S. myrtilloides* is considerable and lies in its role as a habitat-indicator species. Its decline can signal habitat degradation, fragmentation, and accelerating vegetation succession. *S. myrtilloides* serves as a keystone host-plant genus for insect herbivores and plays a crucial role in habitat structure in peatlands, by providing niche habitats for other species and influencing nutrient cycling^28,29^.

Only a few studies provide data on the ecology of this boreal shrub and also on the current status of existing populations. *S. myrtilloides* is a species exposed to threats, primarily habitat isolation and habitat destruction caused by the rapid outgrowth of willows and birches spreading as a result of changes in water regime^19,30,31^. A serious threat to *S. myrtilloides* is spontaneous hybridization with indigenous species of the genus *Salix* (including *S. aurita*)^13^, fungal pathogens^32^ and climate change^33^. Although, it is listed among the plant species considered important in the continent but has inadequate protection status in many countries^34^.

Due to the shrinkage of peatlands and the rapid decline of boreal species in Central Europe over the past few decades, reintroduction is becoming one of the essential tools for restoring extinct populations and for strengthening populations of extremely low abundance found in the wild^35^. It is a handy tool, especially for populations at the edge of geographic ranges. Lack or scarcity of propagules in isolated populations can lead to monoclonal populations with reduced or absent sexual reproduction, significantly limiting their persistence^36^.

The advantage of the deliberate translocation of individuals of a species to its natural range from which it has been lost is that it does not disturb the natural distribution of taxa. For this reason, reintroduction is one of the more common methods used in numerous countries to save endangered plant species^37^. However, such method was rarely applied for the *Salix* species. Most projects were implemented to restore or augment existing *Salix lapponum* populations^38–42^. Similar activities were carried out for *Salix pentandra*, which is rare in the Balkans^43^.

Research presenting the effects of the reintroduction of *S. myrtilloides* is scarce^44^. Most studies on the active protection of this species mainly assess the status of endangered populations and conditions for reintroduction^19,24,44^. Our study is one of the few to evaluate the effectiveness of *S. myrtilloides* restoration based on several years of monitoring of the reintroduced population. Given the research gaps and the need for practical implications for restoring extinct populations, this paper aimed to answer the following questions: What is the survival rate of planted seedlings of *S. myrtilloides* and their fitness after a few years of implementation in the wild? To what extent do microhabitat conditions affect seedling growth? Does the coverage and height of the herb layer affect shoot height and flowering of *S. myrtilloides*? We expected water and soil conditions to have a significant impact on restoration success due to the high diversity of habitats within the quaking bog surrounding the lake. We also assumed that the effectiveness of *S. myrtilloides* restoration may depend on the coverage of competing species.

## Results

### Number of individuals

The success of the restoration, measured by the number of individuals that survived after planting and took up growth, was about 102% after three years—in 2022 a total of 245 individuals were recorded in all aggregations. Four aggregations showed an increase in the number of specimens, another four aggregations showed a decrease, and one cluster showed no change (Table 1). Changes in the number of individuals in clusters ranged from 2% to almost 58% in plus or minus from the initial state. In 2023, the number of individuals in the introduced population survived virtually unchanged, while in the fifth year of the experiment (2024), an increase in the number of living individuals was found in the entire *S. myrtilloides* population to 272 (restoration success rate was 113%). In six of the nine clusters, the success of restoration was more than 100%; in the remaining clusters, it was 71–92%. The dynamics of this parameter in individual aggregations varied, but only in two aggregations (18, 19) was higher (Table 1).

**Table 1:** The success of the restoration of *Salix myrtilloides* between 2019 and 2024 by Lake Wiejki as measured by the number of individuals (Ni) and difference (%) over consecutive study periods.

Cluster number	2019	2022	2023	2024
	Ni	%	%	%
9	24	116.7	100	112.5
10	48	97.9	91.7	91.7
11	24	100	66.7	70.8
12	24	137.5	104.2	125
13	24	95.8	154.2	150
14	24	120.8	108.3	120.8
15	24	83.3	129.2	133.3
18	24	41.7	54.2	158.3
19	24	129.2	112.5	79.2
Total	240	102.1	101.3	113.3

For the restoration year (2019), the numbers of individuals resettled in nine aggregations are given.

### Height of aboveground shoots

**Fig. 1:**
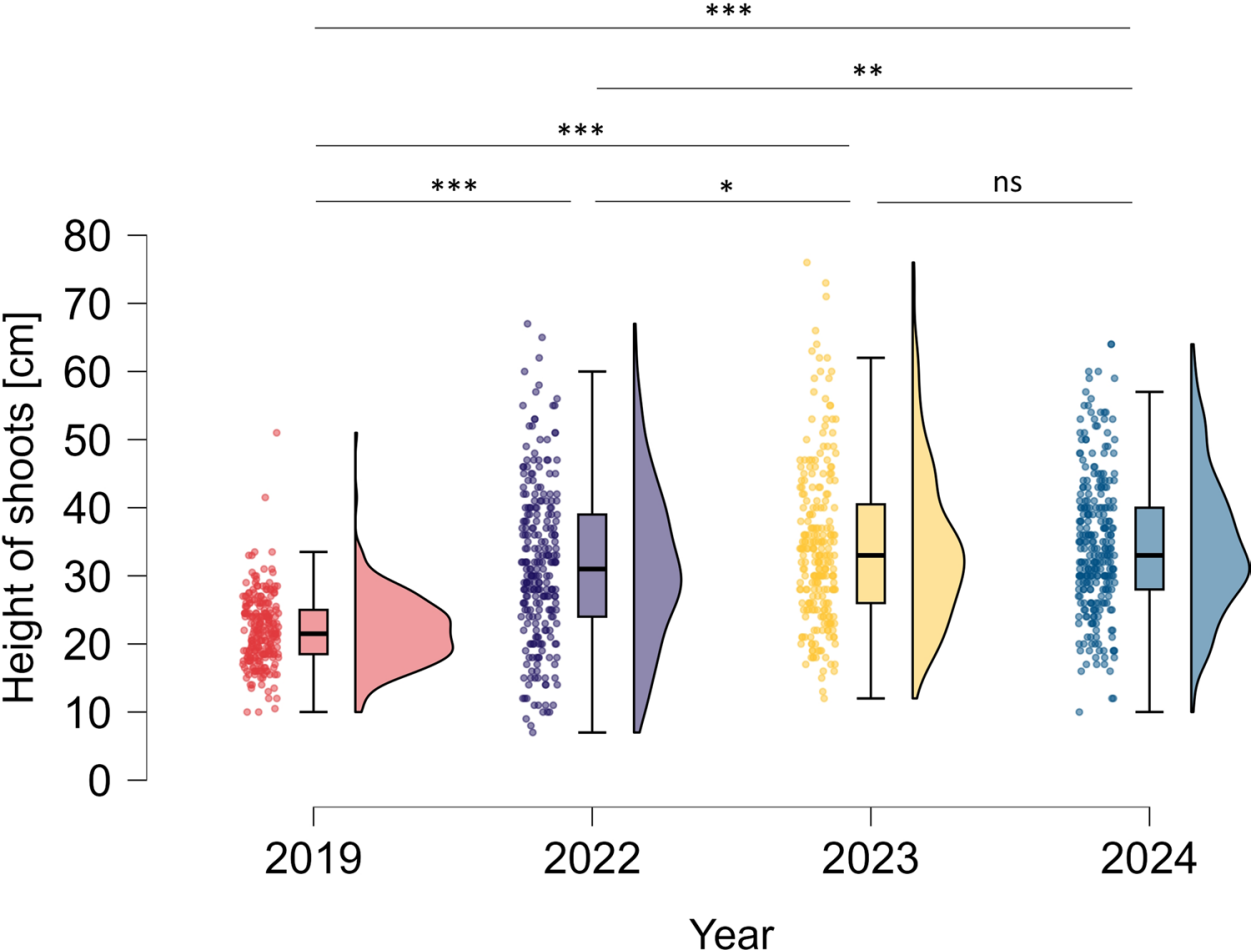
Changes in the height of *Salix myrtilloides* shoots. Asterisks denote significant differences in the Kruskal–Wallis test followed by Dunn’s post hoc tests: ***p < 0.001; **p < 0.01; *p < 0.05; *ns* not significant p > 0.05.

At the beginning of the experiment (2019), the height of introduced *S. myrtilloides* seedlings ranged from 10 to 51 cm and averaged 21.98 ± 5.16 cm (mean ± SD). Within individual clusters the average height was similar and ranged between 19.5 ± 2.89 cm (cluster 11) and 23.63 ± 5.27 (cluster 9). Three years after planting, in 2022, we found the average length of shoots significantly bigger (31.56 ± 11.70 cm; p < 0.001; Kruskall–Wallis test, Fig. 1). The lowest shoots were recorded in cluster 11 (average 21.17 ± 5.86 cm), while the highest shoots were observed in clusters 10 (average 37.15 ± 14.29 cm) and 9 (36.14 ± 13.05 cm). In subsequent seasons, aboveground shoots of *S. myrtilloides* reached sizes a few centimeters higher (average 34.29 ± 11.70 cm in 2023 and 34.53 ± 10.25 cm in 2024), without a significant differences between the last two seasons (2023 and 2024). Finally, after 5 years the aboveground shoots of *S. myrtilloides* increased their length by an average of 12.55 cm. Height measurements of all *S. myrtilloides* individuals are given in the Supplementary Table S1.

At the initial stage of the study (2022), no correlations were found between the height of aboveground shoots of *S. myrtilloides* and the vegetation cover within the aggregation. However, in 2024, five years after planting the seedlings, significant correlations were found between the average height of *S. myrtilloides* shoots and the average height of the herb layer (Spearman’s coefficient 0.867, p < 0.005) and between the average height of *S. myrtilloides* shoots and the total cover of nine competing plant species within aggregations (Spearman’s coefficient 0.729, p < 0.05; Supplementary Table S2).

### Flowering efficiency

In 2022 generative shoots were observed in all nine clusters (Supplementary Table S1). In seven clusters, flowers were recorded on 11–17 individuals per 24/48 seeded and only two clusters had fewer flowering individuals (1–6). In total, the proportion of flowering individuals was 40.82% (100 out of 245 individuals; Table 2). In the following season (2023), only 64 out of 243 individuals (26.34%) took up flowering, and in the last year of the study (2024), 66 individuals out of 272 forming the local population were flowering (24.27%). Overall, after five years of the experiment, generative shoots were present within seven of the nine clusters established at the beginning of the study. No significant differences in the average number of flowering shoots of *S. myrtilloides* were found between individual aggregations. Significant differences in flowering were seen between groups in certain years. The year 2022 saw the highest proportion of flowering shoots in aggregations 11 to 19. In subsequent years (except for cluster 18), flowering in these aggregations was lower.

**Table 2:** The success of *Salix myrtilloides* restoration performed in 2019–2024 at nine sites by Lake Wiejki as measured by the number of individuals that produced inflorescences in subsequent years of the experiment

Cluster number	2019	2022		2023		2024	
	Ni	Nr||Nf	%	Nr|Nf	%	Nr|Nf	%
9	24	28|10	35.71	24|3	12.50	27|14	51.85
10	48	47|17	36.17	44|20	45.45	44|14	31.82
11	24	24|14	58.33	16|8	50	17|6	35.29
12	24	33|15	45.45	25|7	28	30|7	23.33
13	24	23|11	47.83	37|6	16.22	36|6	16.67
14	24	29|13	44.83	26|4	15.38	29|0	0
15	24	20|6	30	31|6	19.35	32|0	0
18	24	10|1	10	13|5	38.46	38|12	31.58
19	24	31|13	41.94	27|5	18.52	19|7	36.84
Total	240	245|100	40.82	243|64	26.34	272|66	24.27

*Ni* number of individuals introduced, *Nr* number of individuals recorded in a given year, *Nf* number of flowering individuals in a given year.

### Assessment of microhabitat conditions

The soil solution was slightly acidic, with a pH of around 5.0 and a low concentration of dissolved substances, measured as an electrical conductivity (EC) that ranged between 90 and 140 μS·cm^–1^ (Table 3, Supplementary Table S3). The calcium concentration did not exceed 30 mg·dm^–3^, and the magnesium concentration was below 5 mg·dm^–3^. The total phosphorus (P_tot_) and P–PO_4_^–3^ content were unexpectedly high, reaching up to 5 mg·dm^–3^ in the hollows in plots 12, 13, and 15. The soil solution was rich in total nitrogen, ranging from approximately 12 to 21 mg·dm^–3^. N–NH_4_^+^ reached 0.9 mg·dm^–3^, and N–NO_3_^–^ concentration ranged between 0.08 and 0.23 mg·dm^-3^.

**Table 3:** Microhabitats, water level and physicochemical properties of acrotelm water in Lake Wiejki concerning the restoration success rate of *Salix myrtilloides*

Parameter	Clusters 18, 19	Clusters 9, 10	Clusters 12, 13, 15
Microhabitat	Humock	Humock	Hollow
Average depth water table in 2024 [m b.g.l.]	0.29	0.13	0.05
pH	4.92–5.18	5.12–5.24	4.65–5.02
EC [μS·cm^-1^]	113–117	118–137	88–110
N-NO_3_ [mgN·dm^-3^]	0.084–0.085	0.088–0.231	0.081–0.086
N-NH_4_ [mgN·dm^-3^]	0.394–0.553	0.612–0.870	0.521–0.855
N_tot_ [mgN·dm^-3^]	15.54–17.00	11.71–21.12	11.81–17.31
P-PO_4_ [mgP·dm^-3^]	0.73–0.96	0.86–1.44	1.92–4.72
P_tot_ [mgP·dm^-3^]	1.50–1.53	1.58–1.99	2.80–4.88
Na [mg·dm^-3^]	9.65–10.04	7.57–12.29	9.78–10.62
Ca [mg·dm^-3^]	23.01–24.62	26.28–31.23	17.02–25.24
Mg [mg·dm^-3^]	3.07–3.12	2.78–4.00	2.06–2.62
TOC [mg·dm^-3^]	127.0–131.8	100.1–128.6	93.8–151.6
Restoration success rate			
Change in the number of individuals [%]	79–152	92–112	125–150
Flowering efficiency [%]	32–37	32–52	0–23

*EC* Electrical conductivity, *TOC* total organic carbon.

**Fig. 2:**
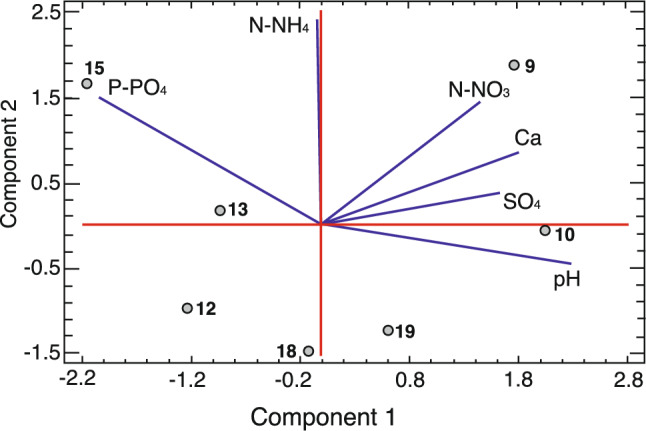
Biplots displaying the aggregations of *Salix myrtilloides* (numbered points) and the variables characterizing soil solution (the ends of the solid blue lines).

PCA extracted three components that together accounted for 90.8% of the variability in the original data (Fig. 2). Component 1 (40.9%) was negatively correlated with orthophosphate concentration and positively correlated with pH. Component 2 (30.5%) showed positive correlations with ammonium and orthophosphate concentrations, while component 3 (19.4%) was positively correlated with sulfate and calcium and negatively correlated with pH and nitrate concentrations.

The aggregations 10, 9, and 19, which were characterized by the most successful shoot flowering, were most strongly associated with component 1. Flowering was the weakest on the most acidic site (component 2), which was additionally distinguished by its high phosphorus content (15).

## Discussion

In recent years, due to the scarcity and isolation of suitable habitats, the possibility of dispersal of relict boreal species in Central Europe drastically decreased^24^, causing a dramatic threat of extinction of local populations^19^. Thus, reintroduction or strengthening wild populations of endangered plant species becomes a priority, involving the responsibility of individual countries to maintain specific taxa on a continental scale. Based on the experience gained from completed species reintroduction projects^37^, their final success can be controlled by numerous factors, such as habitat features, competition from other species, inadequate habitat selection, misidentification of the causes of population decline, type and quality of propagules, techniques for planting propagules, insufficient numbers of reintroduced individuals. According to Godefroid et al.^37^, only three factors significantly impact the reintroduction’s success: the plant material’s origin and quality, removal of potential competitors, and choosing an appropriate location and habitat.

Two factors are considered the most reliable measure of the success of endangered plant species reintroduction: the survival rate of individuals and the ability of reintroduced individuals to bloom and produce fruits^35,37^. The abundance of the reintroduced population of *S. myrtilloides* in L. Wiejki reserve increased by 13.3% over 5 years relative to the initial state (272 individuals vs. 240 seeded). Only in three aggregations, the survival rate of individuals was slightly lower (71–92% of the state), while in six aggregations, the number of individuals increased by 12–58%. Godefroid et al.^37^, analyzing the effects of numerous population restoration projects on endangered plant species worldwide, found that the average survival rate of seedlings was approximately 52%. The result of our experiment significantly exceeds this value. Although the translocation of endangered species is often reported in the literature, there has been only one attempt to reintroduce *S. myrtilloides*^44^ until now. In this report, authors found the survival rate of individuals one year after planting the seedlings to be 16–50% of the total 48 individuals planted during conservation procedures. In the case of *S. lapponum*, another boreal shrub species similar to *S. myrtilloides* in terms of biology and environmental requirements, the survival rate of reintroduced individuals was 19%-52% (after 7 years^45^, 43–100% (after 1 year^39^, 27–67% (after 1 year^44^, and 56–85% (after 3 years^41^.

The central difficulty that could have affected the development of our project was the acquisition of substantial quantities of *S. myrtilloides* seedlings. By their functions, botanical gardens are an appropriate provider of propagules of endangered plant species. Acquiring propagules from this source would not disturb the weakened populations existing in the wild. However, using endangered species collections held in Polish botanical gardens in population restoration projects can be troublesome, as most consist of a small number of individuals, often collected from mother plants of unknown or uncertain origin. We believe that we have achieved high efficiency of restoration due to the location of the project in a site of high conservation status (nature reserve) after careful analysis of edaphic conditions^37,46^ and also due to the use of an effective and efficient method of obtaining high-quality seedlings through micro-propagation in tissue cultures. The in vitro propagation techniques for seedling production were described in detail in numerous papers^26,27,39,40^, and their effectiveness in projects to restore genetic resources of endangered plant species was rated high^47^. The parental plants from which the propagation material was taken came from a stable population characterized by high genetic diversity^26^ that is considered one of the basic conditions for successful reintroduction^48^.

The high efficiency of plant species reintroduction is usually associated with many individuals used for the introduction. Usually, ~ 400 individuals were applied^37^, and the survival rate of seedlings significantly increased in experiments in which more than a thousand individuals were used. The restoration of *S. myrtilloides* in the L. Wiejki Reserve was based on only 270 seedlings, but the experiment’s success could be considered satisfactory. Although plant dieback, the abundance of the entire population increased relative to the initial state.

We found that the introduced *S. myrtilloides* population was affected by herbivore pressure. Each year, within all clusters, we recorded shoot browsing and numerous shoot damages caused by trampling by moos. Aggregations No. 18 (in 2022–2023) and No. 11 (in 2023–2024) suffered in particular. Many shoots were browsed to about half their height. This phenomenon is relatively common in Scandinavian boreal ecosystems^4^, especially regarding young plants^49^. For boreal willow species (e.g., *Salix phylicifolia*, *Salix glauca*), reindeer can reduce plant height and accelerate shoot dieback by up to 50%^49^ and change plant morphology to a stunted growth form^50^. We supposed that browsing may weaken shoots and increase their susceptibility to frost, even in cold-tolerant boreal willow species. This occurs primarily during Central Europe’s increasingly common snowless winters when these shrubs are not sheltered by snow. Moreover, we speculate that browsing may have stimulated the vegetative growth of *S. myrtilloides* and increased production of new ramets within some clusters. This mechanism likely led to a significant increase in the number of ramets in 2024 in cluster No. 18, following the browsing of numerous shoots in previous years (Table 1). One of the strategies plants use against herbivores is a well-developed regrowth capacity with a poor defense mechanism^51^. The results of our experiment suggest that *S. myrtilloides* may employ such a strategy. This shrub species reproduces mainly vegetatively under natural conditions. However, the possibility of generative reproduction within Polish populations is confirmed by high intra-population genetic diversity in this species as well as high pollen viability and seed germination ability^25–27^. As confirmed, the production of catkins does not guarantee effective generative reproduction. Our observations have found no *S. myrtiloides* seedlings, and all new individuals came exclusively from vegetative reproduction. It cannot be ruled out that genets emerged in the L. Wiejki Reserve population a few years after planting the seedlings. However, this issue needs to be clarified through genetic studies.

Flowering rates are considered one of the essential measures of reintroduction success, and most conservation treatments performed for endangered species are located at a relatively low level of less than 20%^37^. The authors of the only study, including the reintroduction of *S. myrtilloides*^44^, reported that one year after planting seedlings, 30% of the surviving plants produced inflorescences. During our study, we stated that the proportion of flowering plants was even higher, reaching 41% in the third year after planting seedlings. Unfortunately, in the following two years, we recorded a significant decrease in the proportion of plants producing flowers to 24–26%. It cannot be ruled out that the main reason for such substantial changes was the intense pressure of herbivores. The downward trend of this indicator over time is also reported by the authors of numerous other projects carried out around the world^37^. Moreover, we think that a considerable threat to the sustainability of this population may be the presence of numerous willow species overgrowing the peatlands around the lake. *S. myrtilloides* interbreed pretty easily with many species of *Salix*, especially with *Salix aurita*, forming the interspecific hybrid *Salix* x *onusta*^13^. Churski & Danielewicz^19^ reported that such a process can lead to population disappearance. Inter-mixing with more common willow taxa and perpetuating poor genetic diversity is now seriously threatening all relict willows^42^.

**Fig. 3:**
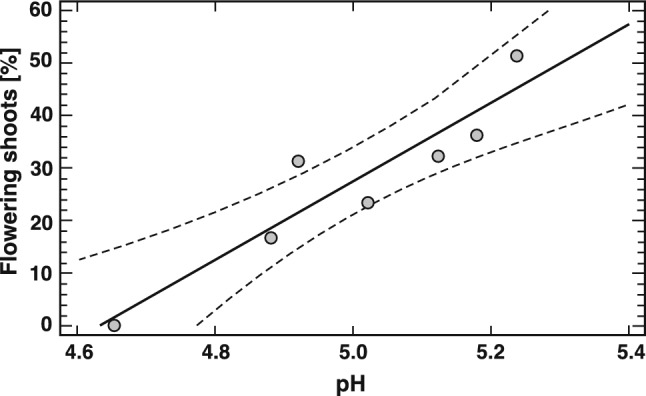
Relationship between the percentage of flowering shoots within aggregations of *Salix myrtilloides* and the acrotelm water pH value.

The chemical composition of acrotelm water varies greatly, which is surprising considering that the probes were taken from a relatively homogeneous patch of peatland. The chemical parameter that most distinguished the sites and was strongly correlated with the introduced plants’ traits- demonstrating the experiment’s success—was pH. There was a linear relationship between the percentage of flowering shoots and the water pH, with a correlation coefficient r = 0.93 at p < 0.01 (Fig. 3). No flowering individuals were observed at a pH of approximately 4.6, whereas at a pH exceeding 5.2, up to 50% of the shoots flowered. An inverse relationship was also found between the number of flowering shoots and the orthophosphate concentration, with a correlation coefficient of r = – 0.83 at p < 0.05. However, due to the limited sample size (n = 7), the results should be treated with caution.

The differences between samples were significant, even for plots just a few meters apart, indicating the vital role of microhabitats (e.g., hummock vs. hollow) in influencing the growth conditions for *S. myrtilloides*. Due to the limited sample size (n = 7), the results should be interpreted cautiously. The small sample size resulted from challenges in obtaining sufficient material, stemming from the specific nature of the studied environment and logistical constraints. Despite these limitations, the statistical analyses revealed potential relationships, providing a basis for future, more comprehensive studies.

Recognition of the determinants of the development and sustainability of *S. myrtilloides* populations is currently very sparse. This is due to the scarcity of natural, unaltered habitats of *S. myrtilloides*, the generally small size of the remaining populations (a few to several dozen individuals) and poor recognition of the localities of this inconspicuous species in countries where it has RTE status.

Lower pH favoured vegetative regrowth (aggregations No. 12, 13, 14, 15) but limited flowering. A higher number of flowering individuals was recorded in the northern part, where pH was slightly higher (aggregations No. 9, 10, 11, 18, 19). An important factor limiting the flowering of *S. myrtilloides* may be the continued heavy watering of the acrotelm. Individuals reintroduced in the hollow, where the peat was continuously saturated, could reproduce vegetatively, but generative reproduction was limited. Moreover, hollows were dominated by *Sphagnum* mosses that can acidify the soil by exchanging protons for basic cations in soil water^52^. It is worth noting that in 2023–2024, Poland faced a severe hydrological drought. Reduced groundwater levels relative to the multi-year average may have significantly impacted the results.

Based on our observations, we conclude that *S. myrtilloides* can survive in new locations if the habitat conditions strictly fall within the spectrum of ecological requirements of this species. The promising results of *S. lapponum* reintroduction carried out in recent years in the Knyszyn Forest^41^ provide grounds for such a conclusion.

## Conclusions

*S. myrtilloides* occurs in Poland in widely dispersed isolated locations, limiting the free flow of genes between populations, dispersal of this species to further distances and colonization of new habitats. Five years after the introduction of swamp willow to L. Wiejki Reserve, the population increased, and flowers appeared in about 24% of the individuals. Nevertheless, its fate in the long term is difficult to predict, mainly because the study period was short.

Microhabitats can be a significant factor in the success of restoration. Our results indicated the critical role of water pH, which, with slight differences, determines individuals’ production of vegetative sprouts and flowering. The significant dynamics in the abundance and flowering efficiency of *S. myrtilloides* individuals, which we observed in individual aggregations in subsequent years, can also be linked to herbivore pressure.

We found that the height of the herbaceous layer and the presence of competing species do not affect the development of *S. myrtilloides* seedlings in the initial period after their establishment. This effect only becomes apparent after a few years—in patches dominated by mosses, *S. myrtilloides* individuals were shorter, while in the vicinity of sedges, grasses, and tall herbs, they reached larger sizes. We suspect that the most critical factor determining this relationship is competition for light.

Based on our previous experience gained during the reintroduction of *S. lapponum* in the Knyszyn Forest^41^ to increase the chances of survival of newly introduced endangered boreal shrub species, restoration projects should be combined with other active conservation treatments, such as raising and stabilizing water tables in wetland environments and cutting down competing trees and shrubs. We also believe that only long-term monitoring can provide reliable data on the conditions and survival of reintroduced populations of these species at the limits of their geographic ranges.

## Material and methods

### Study site

**Fig. 4:**
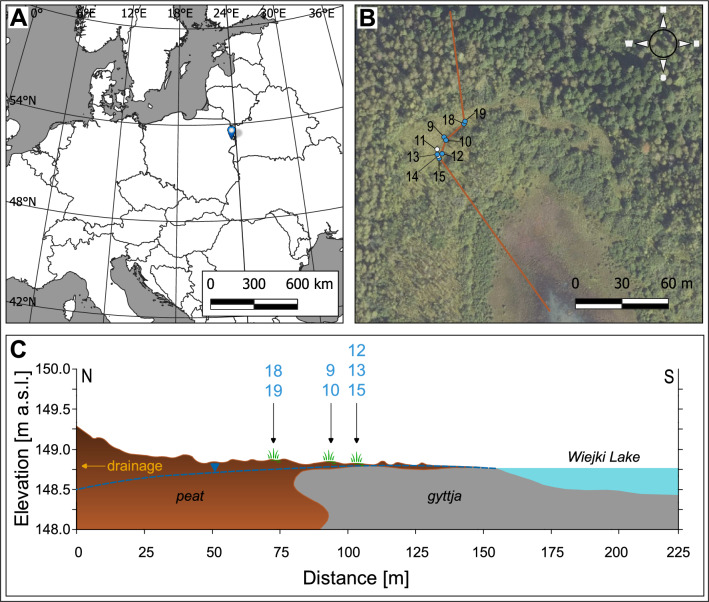
Map of the study site (**A**) and location of restoration plots of *Salix myrtilloides* (**B**); blue points identify the introduction sites with an analysis of the chemical composition of the acrotelm water, white points without analysis, and the brown polyline shows the location of the cross-section on panel C; The variation of the microrelief was developed based on the Digital Terrain Model from a point cloud obtained with LiDAR technology, overview layout of the organic layers is shown after corings with a handle peat sampler Instorf (Eijkelkamp, The Netherlands), and the groundwater level is drawn based on observations made in the vegetation season 2024; Inconsistent plot numbering is due to other restoration plots in the area. Maps were generated using the QGIS 3.28.4-Firenze software (Free and Open Source Software (FOSS); Free Software Foundation, Inc., USA; www.qgis.org). Source for the orthophoto map on panel B: www.geoportal.gov.pl (Terms and conditions: https://www.geoportal.gov.pl/en/about-geoportal/terms-and-conditions/).

We conducted studies in 2019–2024 at Lake Wiejki Reserve, located on the eastern outskirts of the Knyszyn Forest (NE Poland), where a transitional bog typical of *S. myrtilloides* was preserved (Fig. 4).

Lake Wiejki is located in the upper reach of the Supraśl River within a broad peat-filled Gródek-Michałowo depression, formed during the Warta Glaciation^53^. The area is flat, with few hills on the periphery of the basin. At the end of the nineteenth century, slightly south of the lake, a wide canal was dug to drain water from the wetlands into the main river channel. The drainage completed in 1956–57, along with subsequent reclamation works carried out in the 1970s and 1980s, severely impacted the hydrology of this part of the Supraśl R. valley^54^. Despite the disturbances, the zone adjacent to the lake retains well-watered mires with typical zonal distribution of aquatic and marsh vegetation. The littoral zone is dominated by *Equisetum fluviatile*, while the limnetic zone of the lake is occupied by *Ceratophyllum submersi*. The aquatic ecosystems are surrounded by a ring of quaking bogs of variable width dominated by sedges and *Sphagnum* moss. Patches of open moss-sedge communities go into dense thickets with *Salix cinerea*. Decades-old forests with *Betula pubescens, Pinus sylvestris*, and *Alnus glutinosa* surround the entire complex of vegetation overgrowing the quaking bog.

Since 2005, 22.5 hectares of aquatic and wetland habitats have been protected as a nature reserve harboring rare plant species (including *Chara globularis*, *Betula humilis*, *Dactylorrhiza latifolia*, and *Drosera rotundifolia*) and animals (including *Emys orbicularis*). Until the 1990s, a site of *S. lapponum*—one of the glacial relics—existed here. From 2018 to 2020, this species was successfully reintroduced at the site^41^.

The section of the lakeside peatland selected for *S. myrtilloides* restoration was well-watered, well-sunny, and not threatened by tree and shrub invasion. Seedling plantings were planned in the northern part of the peatland within the quaking bogs with *Carex rostrata*, *Carex diandra*, *Menyanthes trifoliata*, *Comarum palustre,* and *Sphagnum* mosses, i.a., *Sphagnum fimbriatum*, *S. teres* and *S. fallax*.

The climate is characterized by warm summers with an average July temperature of 17.9 °C and cool winters with an average January temperature of – 3.9 °C and a shortened growing season of less than 200 days. Annual precipitation in the region averages about 660 mm, with the highest proportion in the summer months (1996–2023^55^. However, the past two decades have seen milder winters with less snow alongside fewer days of rain and higher temperatures, resulting in droughts. Very mild winters and long periods without precipitation resulted in hydrological drought in 2023–2024. From May 1 to June 30, 2024, the average value of the Climatic Water Balance (CWB) was negative and amounted to – 180 mm. The negative CWB continued until September, with values consistently less than – 100 mm^56^. Water conditions in the past few years have been only slightly more favorable than in extremely dry 2024.

### Restoration experiment

For restoration of the *S. myrtilloides* population we used plants obtained through micro-propagation in tissue cultures. The plant material came from one of the largest *S. myrtilloides* population in E Poland located in the Łęczna-Włodawa Lakeland^26^. Swamp willow seedlings used in our experiment were prepared by a team led by Prof. Magdalena Pogorzelec of the Lublin University of Life Sciences as part of the project *Active conservation of endangered relict plant species of the Salicaceae family in peat bog habitats* funded by the European Union through the Infrastructure and Environment Operational Programme (grant no. POIS.02.04.00-00-0008/17). Due to the limited resources of the swamp willow population in Poland, plant material was not deposited in the herbarium. All the necessary permits required by Polish law were obtained before the project activity. In vitro cultivated plants were transferred to a specially prepared substrate mimicking the edaphic conditions of peatland habitats and kept in controlled temperature and humidity for about three months. Then, *S. myrtilloides* rooted seedlings were transferred to specially prepared cold frames to acclimate to similar conditions at natural sites. After few months, the plants were planted in the L. Wiejki Reserve. A similar procedure has been successfully applied to propagate other species of *Salix*^57^.

Individuals of *S. myrtilloides* were planted in aggregations of irregular shapes, with areas ranging from about 2 to 4 m^2^. Plants were introduced into the ground with the entire root ball at 20–30 cm spacing in September and October 2019. A total of 240 individuals of *S. myrtilloides* were planted in nine clusters: eight clusters consisting of 24 individuals + one cluster with 48 individuals (Fig. 4). A few male specimens were introduced among the planted seedlings each time. Before planting, the height of all seedlings was measured.

In 2022, three years after the beginning of the experiment, the number of surviving individuals, the number of flowering ones, and the height of shoots were determined within each aggregation. The measurements were repeated in the summer of 2022, 2023, and 2024 to determine the growth rate of aboveground shoots of *S. myrtilloides*. Moreover, the cover of dominant and competing herbaceous and woody species was estimated and the height of the herbaceous layer was measured in the middle part of the growing season (2022) and its early stage (2024). Each time, 20 surveys were taken within each cluster.

### Groundwater chemistry

Manual measurements of water levels were performed in four wells between June and September 2024. Samples of the shallow groundwater/peat soil solution from the root zone (0–20 cm) were collected only once from seven sites (9, 10, 12, 13, 15, 18, 19) using the Eijkelkamp Agrisearch Equipment vacuum sampler (Giesbeek, the Netherlands). The specific conductance (EC) and pH were determined in the field using a Hach Lange HQ40D measuring device (Hach, USA). Ammonium nitrogen (N–NH₄⁺), nitrate nitrogen (N–NO₃⁻), nitrite nitrogen (N–NO₂⁻), orthophosphate phosphorus (P-PO₄^3^⁻), sulfates (SO₄^2^⁻), chlorides (Cl⁻), fluorides (F⁻), calcium (Ca^2^⁺), magnesium (Mg^2^⁺), sodium (Na⁺), potassium (K⁺), and lithium (Li⁺) were analyzed using a compact ICS-1100 ion chromatograph (Dionex, USA) equipped with an isocratic dual-piston pump, vacuum degasser for eluents, thermostatted columns and detector cell, electronic suppression, and a Dionex AS-DV autosampler controlled by Chromeleon 7 software. Total organic carbon (TOC) and total nitrogen (TN) were determined via catalytic combustion at high temperatures using a TOC-L analyzer (Shimadzu, Japan). After mineralization, total phosphorus (TP) was analyzed using the molybdenum blue method.

### Data analysis

The significance of differences between the height of *S. myrtilloides* aboveground shoots in the following years of the experiment (Supplementary Table S1) was tested by one-way analysis of variance (ANOVA) and the Kruskal–Wallis test followed by Dunn’s post hoc tests. Levene’s test examined the homogeneity of the variance of the samples, while the normal distribution of the collected data was tested with the Shapiro–Wilk test at the significance level of p < 0.05. Correlations between population/individual parameters of *S. myrtilloides* and habitat properties and vegetation cover were assessed using Spearman’s and Pearson’s correlation coefficients, which evaluate the strength and direction of linear relationships between variables (p < 0.05). Correlations between the average height of *S. myrtilloides* shoots and the average height of herb layer were determined based on 20 measurements taken within clusters (Supplementary Table S2). Moreover, correlations were assessed between the average height of *S. myrtilloides* shoots and the total cover of nine plant species that compete with this shrub within aggregations (Supplementary Table S2). Statistical analyses were conducted using JASP software (JASP Team, Version 0.19.0). The contingency table (chi-square test) was used to test a difference in flowering of *S. myrtilloides* between aggregations.

Six variables characterizing the habitat’s chemical composition were used for the principal component analysis (PCA): N–NH_4_^+^, SO_4_^2–^, Ca^2+^, pH, N–NO_3_^-^, and P–PO_4_^3–^. Two variables, SO_4_^2–^ and P–PO_4_^3–^, were logarithmized to obtain a distribution more similar to a normal distribution. All variables were standardized. The analysis was performed in Statgraphics Centurion 18.

## Supplementary Information

Supplementary Information 1.

Supplementary Information 2.

Supplementary Information 3.

## Data Availability

Data is provided within the manuscript or supplementary information files.
